# Development and prospective validation of a multitarget urine RNA assay for noninvasive detection of urothelial carcinoma

**DOI:** 10.1016/j.isci.2025.114154

**Published:** 2025-11-19

**Authors:** Hua Xu, Shuai Wang, Dingwei Xue, Zhihui Xu, Qi Zhang, Qijun Wo, Banggao Huang, Zujie Mao, Feng Liu, Shuixin Lou, Jie Yuan, Shibin Zhu, Lifeng Ding, Yixin Wo, Zhengzhi Luo, Jinying Chen, Qinghua Xu, Gonghui Li, Xiaolong Qi, Dingwei Ye

**Affiliations:** 1 Department of Urology, Fudan University Shanghai Cancer Center, Fudan University, No. 270, Dong’an Road, Xuhui District, Shanghai 200032, China; 2 Department of Oncology, Shanghai Medical College, Fudan University, No. 270, Dong’an Road, Xuhui District, Shanghai 200032, China; 3 Urology & Nephrology Center, Department of Urology, Zhejiang Provincial People’s Hospital, Affiliated People’s Hospital, Hangzhou Medical College, No. 158, Shangtang Road, Hangzhou 310000, China; 4 Department of Urology, Sir Run Run Shaw Hospital, Zhejiang University, School of Medicine, No. 3, Qingchun East Road, Shangcheng District, Hangzhou 310000, China; 5 The Canhelp Genomics Research Center, Canhelp Genomics Co., Ltd., No. 22, Xinyan Road, Linping District, Hangzhou 310000, China; 6 Department of Integrative Oncology, Fudan University Shanghai Cancer Center, Fudan University, No. 270, Dong’an Road, Xuhui District, Shanghai 200032, China; 7 Shanghai Key Laboratory of Medical Epigenetics, Institutes of Biomedical Sciences, Fudan University, No. 130, Dong'an Road, Xuhui District, Shanghai 200032, China

**Keywords:** Diagnostic procedure, Diagnostic technique in health technology, Cancer

## Abstract

Accurate and noninvasive diagnosis is critical in the management of urothelial carcinoma (UC). This study develops and prospectively validates a multitarget urine RNA (mt-uRNA) test for noninvasive UC detection. The RNA panel, built using quantitative RT-PCR and support vector machine algorithms, achieved an area under the curve of 0.956 in the upgraded training set of 688 samples. In the prospective validation phase, urine samples from 752 patients in three Chinese medical centers were analyzed, and the test achieved an overall accuracy of 93.4%, with a sensitivity of 92.3% and specificity of 94.1%. This robustness was maintained across subgroups, demonstrating high sensitivity for carcinoma *in situ*, low-grade, and upper tract UC (83.3%, 89.9%, and 97.3%) and high accuracy in hematuria, residual and recurrent subpopulations (93.6%, 86.9%, and 92.7%). These findings underscore the mt-uRNA test’s high diagnostic utility for noninvasive UC detection, offering a promising alternative/adjunct to endoscopy for hematuria investigations and surveillance.

## Highlights

• A multitarget urine-based RNA test enables noninvasive urothelial carcinoma detection
• Prospective multicentric validation confirms its high diagnostic accuracy
• Robustness is demonstrated across diverse patient subgroups
• The test offers a diagnostic alternative/adjunct in urothelial carcinoma management

## Graphical abstract

**Figure:**
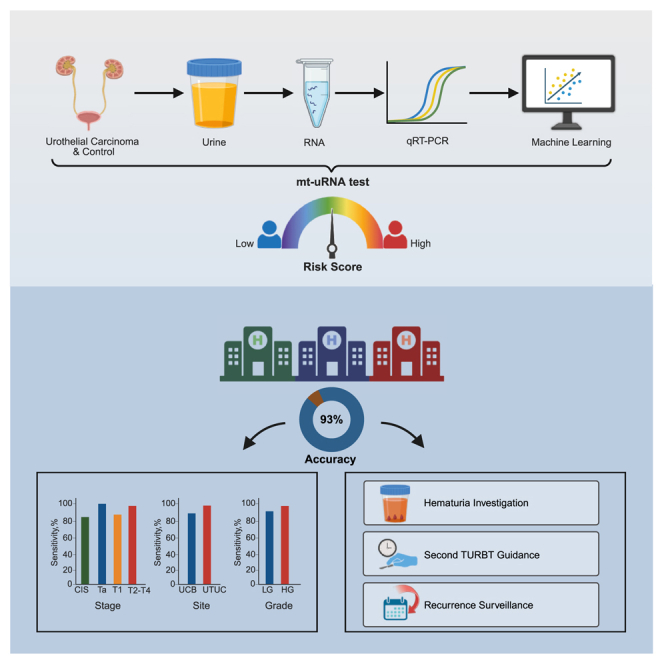

## Introduction

Urothelial carcinoma (UC), which includes upper urinary tract urothelial carcinoma (UTUC) and urothelial carcinoma of the bladder (UCB), has risen to rank as the ninth most common malignancy worldwide.^1^ UCB accounts for 90%–95% of UC, while UTUC is uncommon and makes up only 5%–10%.^2^ Patients with advanced-stage UC have a median overall survival of 15 months,^3^ making early diagnosis and relapse surveillance particularly important for individuals at high risk or receiving surgery.

Endoscopy and biopsy are the current gold standard for UC diagnosis and surveillance. However, the use of endoscopy may miss lesions that are in low grade (LG) and carcinoma *in situ* (CIS).^4^ Meanwhile, the invasive examinations cause patient suffering and increase the risk of infection and tumor recurrence.^5^ Cytology, the commonly applied noninvasive alternative, has an overall specificity of 85%–100% for UC,^6^^,^^7^ but the sensitivity for LG lesions remains only 17%.^8^

Urinary biomarkers targeting DNA, RNA, and proteins were developed as valuable alternatives or adjuncts to endoscopy for the detection of UC. Protein-based markers (such as nuclear matrix protein 22 [NMP22]) are currently used in clinical practice,^7^^,^^9^ but the performance is susceptible to benign conditions such as urinary tract infection (UTI) and calculus, and the accuracy is poor in case of LG tumors.^10^ DNA-based tests, such as DNA methylation, DNA mutations, and copy number alteration profiling, have been reported to exhibit a sensitivity range of 80%–91% and a specificity range of 73%–95%.^11^^,^^12^^,^^13^^,^^14^^,^^15^ However, there is still much more room for improvement in detection and surveillance of UC, as well as high-quality evidence from prospective trials.

Multitarget RNA biomarkers have emerged as a promising alternative methodology for UC detection. Urine-based RNA tests, utilizing gene panels ranging from 4 to 32, have been proven to demonstrate improved diagnostic sensitivity when compared to cytology or NMP22 assay.^16^^,^^17^ In addition, RNA-based tests provide enhanced overall sensitivities and specificities for detecting both LG and high grade (HG) tumors,^18^^,^^19^ compensating for the deficiencies of the low sensitivity for LG lesions of cystoscopy and cytology.

In our previous work, we developed a multi-gene expression test for the noninvasive detection of UCB, which achieved an overall accuracy of 89.91%.^20^ Notably, 17% cases of UTUC also have concurrent UCB, and UCB recurrence occurs in approximately 22%–47% of all cases of UTUC after surgery.^21^ We subsequently identified a multitarget RNA test for UTUC, which showed robust performance with 89.38% accuracy.^22^ In this study, we advanced our work further by developing and validating a multitarget urine RNA (mt-uRNA) test to detect both UCB and UTUC, offering a promising approach for the noninvasive detection and surveillance of UC.

## Results

**Figure 1:**
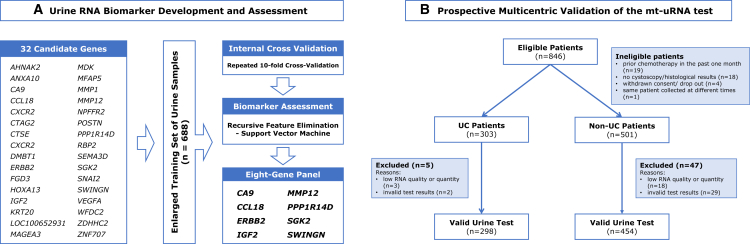
Workflow for the development and validation of the multitarget urine RNA (mt-uRNA) test (A) Urine RNA biomarker development and assessment: the training cohort was established by pooling all available samples from previous studies, resulting in a total of 688 samples. An eight-gene panel was identified from 32 candidates using recursive feature elimination-support vector machine with ten-fold cross-validation. (B) Prospective multicentric validation of the mt-uRNA test: a total of 846 subjects were initially enrolled in the study. Among them, 42 were deemed ineligible due to prior chemotherapy within one month (*n* = 19), lack of cystoscopy/histological results (*n* = 18), withdrawal of informed consent or drop out (*n* = 4), and sample resampling (*n* = 1), thus 303 UC patients and 501 controls remained. Subsequently, 5 UC samples and 47 controls were excluded from the final analysis. Reasons for exclusion included low RNA quality or quantity (3 UCs and 18 controls) and invalid test results (2 UCs and 29 controls). Ultimately, 298 UC patients and 454 controls were included in the final validation analysis to confirm the performance of the mt-uRNA test. Abbreviation: mt-uRNA, multitarget urine RNA; UC, urothelial carcinoma.

As depicted in Figure 1, we developed a two-phase protocol with independent cohorts: the development phase of mt-uRNA test with a case-control cohort of 688 samples, followed by a clinical validation phase within a multicentric, prospective trial of 846 participants. To our knowledge, this is the most comprehensive clinical study to date focused on the urine RNA biomarkers for UC detection.

### Urine RNA biomarker development and assessment

The upgraded training set comprised a total of 688 urine samples. Among these, 328 samples (47.7%) were confirmed to be UC positive, which included UCB (*n* = 267) and UTUC (*n* = 61). We employed the support vector machine-recursive feature elimination (SVM-RFE), to efficiently identify a minimal set of genes that maintains optimal classification accuracy. This approach ensures that the selected genes are not only the most informative but also the least in number. SVM-RFE was implemented using the caret package in R with a linear kernel, and the penalty factor (C) was set to 0.01. Utilizing a 10-fold cross-validation (CV) process repeated 100 times, the algorithm selected candidate features and trained SVM classifiers on 90% of the samples while reserving 10% for testing in each iteration. We then used a consensus method to assess the reliability of the selected genes across the 10-fold CV process. The appearance rate of each gene selected among the 100 iterations was ranked, allowing us to identify the most consistently selected genes. The eight genes (*CA9, CCL18, ERBB2, IGF2, MMP12, PPP1R14D, SGK2,* and *SWINGN*) showing the highest appearance rates were selected, indicating their robustness across different data splits. For each test specimen, the trained model analyzed the multi-RNA expression profiles to generate a risk score which indicates the likelihood that the specimen will be confirmed as UC. The eight-gene model achieved an area under the curve (AUC) value of 0.956 in the complete training set. A cut-off risk score of 50 was established using Youden’s J statistic to optimize the balance between sensitivity and specificity.

Table 1 provides an overview of the identified biomarkers, highlighting that four genes were up-regulated while the rest were down-regulated in UC patients relative to controls. Functional enrichment analysis utilizing the ToppGene Suite (https://toppgene.cchmc.org/) uncovered significant associations of these biomarkers with key pathways,^23^ including ‘transitional cell carcinoma’ (*CA9, ERBB2*). A full list of enrichment analysis is detailed in Table S1.

**Table 1:** Description of 8 genes annotation

Gene symbol	Gene description	Cytogenetic band	RNA Type	Regulation^a^
CA9	Carbonic Anhydrase 9	9p13.3	mRNA	Up
CCL18	C-C Motif Chemokine Ligand 18	17q12	mRNA	Down
ERBB2	Erb-B2 Receptor Tyrosine Kinase 2	17q12	mRNA	Down
IGF2	Insulin Like Growth Factor 2	11p15.5	mRNA	Up
MMP12	Matrix Metallopeptidase 12	11q22.2	mRNA	Up
PPP1R14D	Protein Phosphatase 1 Regulatory Inhibitor Subunit 14D	15q15.1	mRNA	Up
SGK2	Serum/Glucocorticoid Regulated Kinase 2	20q13.12	mRNA	Down
SWINGN	SWI/SNF Complex Interacting GAS6 Enhancer Non-Coding RNA	13q34	lncRNA	Down

a Up, log_2_ fold change >1; down, log_2_ fold change < – 1.

### Inter-laboratory/inter-operator reproducibility

Reproducibility was evaluated by three operators performing the pooled samples over a course of five days in three laboratories. The inter-laboratory and inter-operator comparison study showed a 100% overall concordance of mt-uRNA test results across all laboratories and operators. Additionally, desirable coefficient of variation <5% was reached for each of the eight target genes (Figure S1). This experiment demonstrated high inter-laboratory and inter-operator reproducibility, which served as a foundational basis for multicentric validation.

### Prospective multicentric validation

#### Patient enrollment and characteristics

Urine samples from a total of 846 participants were prospectively collected, prior to any invasive tests or treatments. Based on the inclusion and exclusion criteria detailed in the methods section, 94 individuals who did not meet the criteria were excluded. Subsequently, a total of 298 UCs and 454 controls were included for further study (Figure 1). As shown in Table 2, the average age of the controls was 65.5 years (22–99), while the mean age of UC was 67.2 years (35–92). The control group consisted of 382 males (84.1%) and 72 females (15.9%), while the UC group comprised 248 males (83.2%) and 50 females (16.8%). No group differences were found regarding age and gender.

**Table 2:** Patient characteristics of validation cohort

Characteristics	UC (*N* = 298)	Control (*N* = 454)	*p* value^a^
Age			
Mean (SD)	67.2 (10.7)	65.5 (11.3)	0.071
Gender, n (%)			0.82
Male	248 (83.22%)	382 (84.14%)	
Female	50 (16.78%)	72 (15.86%)	
History of UC			
Yes	79 (26.51%)	57 (12.56%)	<0.001
Tumor organ, n (%)			
Bladder	261 (87.58%)	–	–
Renal pelvis	13 (4.36%)	–	–
Ureter	12 (4.03%)	–	–
Multiple^b^	12 (4.03%)	–	–
Tumor stage, n (%)			
CIS	12 (4.03%)	–	–
Ta	12 (4.03%)	–	–
T1	106 (35.57%)	–	–
T2-T4	32 (10.74%)	–	–
N/A	136 (45.64%)	–	–
Tumor grade, n (%)			
LG	69 (23.15%)	–	–
HG	182 (61.07%)	–	–
N/A	47 (15.77%)	–	–
Number of tumors, n (%)^c^			
Solitary	86 (28.86%)	–	–
Multiple	117 (39.26%)	–	–
N/A	95 (31.88%)	–	–
Size of tumors, n (%)^c^			
≤3 cm	150 (50.34%)	–	–
>3 cm	80 (26.85%)	–	–
N/A	68 (22.82%)	–	–
Type of benign disease, n (%)		271 (59.69%)	–
Benign tumor	–	20 (4.41%)	–
Inflammation	–	78 (17.18%)	–
BPH	–	74 (16.30%)	–
Urolithiasis	–	7 (1.54%)	–
Others^d^	–	92 (20.26%)	–
Type of non-UC cancer, n (%)	–	183 (40.31%)	–
Bladder	–	11 (2.42%)	–
Prostate	–	110 (24.23%)	–
Kidney	–	55 (12.11%)	–
Others^e^	–	7 (1.54%)	–

BPH: benign prostatic hyperplasia; CI, confidence interval; CIS: carcinoma *in situ*; HG, high grade; LG, low grade; N/A, not available; SD, standard deviation; UC, urothelial carcinoma; UCB: urothelial carcinoma of the bladder; UTUC: upper urinary tract urothelial carcinoma.

a *p* value was calculated by Wilcoxon Rank-Sum test or Pearson’s Chi-squared test.

b The UTUC cohort (*n* = 37) included 25 UTUC-only patients and 12 patients with synchronous UTUC and concomitant UCB.

c The tumor numbers and tumor sizes were integrated from results of postoperative pathological examination, intraoperative surgical records, and preoperative imaging assessments.

d Other benign diseases included granuloma, cyst, etc.

e Other non-UC cancers included carcinomas of penis, lymphoma, melanoma, etc.

Of the 298 patients with UC, 261 (87.6%) had bladder involvement, 12 (4.0%) had ureter involvement, 13 (4.4%) had renal pelvis involvement, and an additional 12 (4.0%) had involvement in multiple sites. Due to limited sample sizes, UTUC-only and UTUC with concomitant bladder carcinoma were merged into a single group UTUC cohort (*n* = 37).

Besides, 136 patients had a previous history of UC and were enrolled in surveillance analysis. Among this subgroup, 79 experienced recurrence and 57 remained recurrence-free. The proportion of patients with a prior UC history was higher in UC group (*p* < 0.001). Definitive previous instillations of either chemotherapy or Bacillus Calmette Guerin were available for 72 patients (68 with prior treatments, 4 without). There was no significant difference in the proportion of patients receiving additional anti-tumor treatments between the two groups (*p* = 0.62) (Table S2).

#### Performance in overall study population

**Figure 2:**
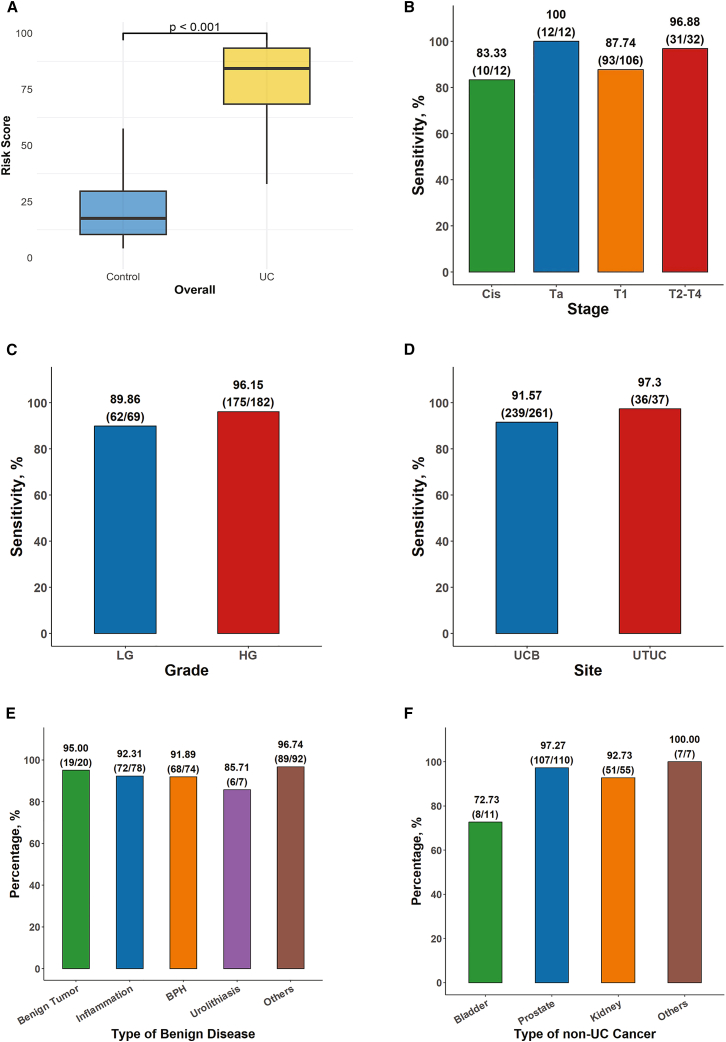
Results of the mt-uRNA test in the multicentric validation study (A–F) Boxplot (A) shows the comparisons of mt-uRNA test-derived risk scores of patients with UCs (*n* = 298) and controls (*n* = 454). The center lines within the box indicate the median, the boxes represent the interquartile range, and the whiskers extend to the minimum and maximum values. Wilcoxon rank-sum test was used to compare difference between groups (*p* < 0.001). Bar graphs show the sensitivity of UC detection by mt-uRNA test in different disease-stage subgroups (B), cancer-grade subgroups (C), or tumor sites (D); and the specificity of the mt-uRNA test for the indicated types of benign diseases (E) and non-UC cancers (F). Bar heights represent the corresponding sensitivity or specificity, calculated as the percentage of true positive/negative cases out of the total number of patients in each subgroup. The numerical values and ratios are presented above each bar. The data presented are descriptive statistics. The exact sample size (n), representing the number of patients, for each subgroup is as follows: (B) CIS (*n* = 12), Ta (*n* = 12), T1 (*n* = 106), and T2–T4 (*n* = 32); (C) LG (*n* = 69) and HG (*n* = 182); (D) UCB (*n* = 261) and UTUC (*n* = 37); (E) benign tumor (*n* = 20), inflammation (*n* = 78), BPH (*n* = 74), urolithiasis (*n* = 7), and other benign diseases (*n* = 92); (F) bladder (*n* = 11), prostate (*n* = 110), kidney (*n* = 55), and other non-UC cancers (*n* = 7). Abbreviation: CIS: carcinoma *in situ*; LG: low grade; HG: high grade; UCB: urothelial carcinoma of the bladder; UTUC: upper urinary tract urothelial carcinoma; BPH: benign prostatic hyperplasia.

Of 752 eligible participants, patients with UC exhibited significantly higher risk scores compared to the controls (*p* < 0.001) (Figure 2A). The overall agreement between the mt-uRNA test classification and the reference diagnosis was 93.35% (95% confidence interval [CI], 91.33–95.03). The sensitivity and specificity reached 92.28% (95% CI, 88.64–95.04) and 94.05% (95% CI, 91.46–96.04), respectively. The positive predictive value (PPV) and negative predictive value (NPV) of mt-uRNA test were 91.06% (95% CI, 87.26–94.03) and 94.89% (95% CI, 94.29–96.73), respectively. As measured by the AUC, the mt-uRNA test reached a value of 0.969 (Table S3).

As illustrated in Figure 2B, the test sensitivity reached 83.33% (10/12) for CIS, 100% (12/12) for stage Ta, and 87.74% (93/106) for stage T1. Meanwhile, the mt-uRNA test achieved 96.88% sensitivity in diagnosing tumors staged T2–T4 (31/32). The test sensitivity was 89.86% (62/69) for LG tumors and 96.15% (175/182) for HG tumors (Figure 2C). Based on tumor locations, the sensitivity was 91.57% (239/261) for UCB and 97.30% (36/37) for UTUC (Figure 2D).

Furthermore, the mt-uRNA test also exhibited a high specificity of 94.05% (427/454) in distinguishing UC from other urologic conditions. Specifically, there were 271 cases of benign diseases, accounting for 59.7% of the control group, and 183 other urinary carcinomas. The specificity was 93.73% (254/271) for patients with benign diseases and 94.54% (173/183) for other urinary carcinomas (Figures 2E and 2F). Notably, among the patients with benign diseases, 92.31% patients with urinary inflammation/infection, 91.89% patients with benign prostatic hyperplasia, 95.00% patients with benign neoplasm, and 85.71% patients with urolithiasis were correctly identified. For other urinary carcinomas, the specificity was 97.27%, 92.73%, and 72.73% for prostate cancers, kidney cancers, and bladder non-UC cancers, respectively. These results confirm that the mt-uRNA test can distinguish both benign and malignant controls effectively.

#### Mt-uRNA test versus routine clinical urine-based tests

Validation specimens with routine clinical urine-based tests (such as urine cytology, fluorescence *in situ* hybridization [FISH] and NMP22), including 66 UC patients and 36 controls, were investigated to compare the routine urine tests to the mt-uRNA test. For the urine-based UC detection tests currently used in clinical practice, the result of the routine testing panel is considered positive if any single test yields a positive result. The mt-uRNA test outperformed the routine testing panel with significantly improved sensitivity (95.45% vs. 36.36%; *p* < 0.001). Specificity remained high with only one control patient giving a positive mt-uRNA result, thereby generating a specificity of 97.22% (95% CI, 85.47–99.93).

#### Performance in hematuria subgroup

**Figure 3:**
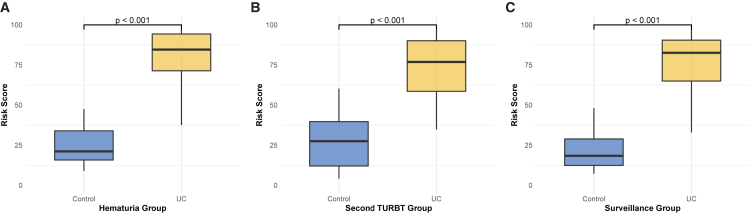
The mt-uRNA test-based risk score in hematuria, residual and recurrent subpopulation (A–C) Boxplots show the comparison of the mt-uRNA test-derived risk scores of UCs and controls in hematuria (A), residual (B) and recurrent subpopulation (C). The exact sample size (*n*), representing the number of patients, for each subgroup is as follows: (A) 172 UC patients and 32 controls, (B) 38 UCs and 23 controls, (C) 79 UCs and 57 controls. For each box, the center line represents the median, the box boundaries represent the interquartile range, and the whiskers extend to the minimum and maximum values. Comparisons in panels A–C were assessed by the Wilcoxon rank-sum test. All differences were statistically significant (*p* < 0.001).

In a subgroup comprising 204 patients presented with hematuria, UC patients have significantly higher risk scores compared to those with other urologic diseases (*p* < 0.001) (Figure 3A). The mt-uRNA test achieved 93.63% accuracy (95% CI, 89.35–96.56), 94.19% sensitivity (95% CI, 89.57–97.18), and 90.63% specificity (95% CI, 74.98–98.02), with an AUC of 0.941 (Table S4). The PPV and NPV of mt-uRNA test were 98.18% (95% CI, 94.78–99.62) and 74.36% (95% CI, 57.87–86.96), respectively.

#### Second TURBT subpopulation

Second transurethral resection of bladder tumor (TURBT) is recommended to be performed within 2–6 weeks for patients with HG and T1 tumors.^4^ However, we still lack effective procedures to estimate if the patient has residual tumors. In our validation cohort, a total of 61 patients were subjected to a second TURBT. Of which, 38 were confirmed to have residual tumors. In the second TURBT subpopulation, patients with residual tumors achieved higher risk scores compared to those without residual disease (*p* < 0.001) (Figure 3B). The mt-uRNA test accurately identified 34 of 38 residual tumors, illustrating a sensitivity of 89.47% (95% CI, 79.20–97.06), whilst 19 residual-free patients were correctly classified by the mt-uRNA test, yielding a specificity of 82.61% (95% CI, 61.22–95.05), with an AUC of 0.949 (Table S5). The PPV and NPV of mt-uRNA test were 89.47% (95% CI, 79.20–97.06) and 82.61% (95% CI, 61.22–95.05), respectively.

#### Performance in recurrence subpopulation

Non-muscle-invasive bladder cancer (NMIBC), accounting for 80% of bladder cancers, has a high recurrence rate, with a one-year recurrence rate of 15%–61%, and a five-year recurrence rate of 31%–78%.^5^ To further evaluate the performance of mt-uRNA test in detecting recurrent tumors, we conducted a subgroup analysis involving 136 patients with a history of UC. Of which, 79 individuals were confirmed to have recurrent tumors through histopathological examination of the resected tumor samples. Patients experienced recurrence showed higher risk scores compared to those who did not experience recurrence (*p* < 0.001) (Figure 3C). The test performance reached 92.65% accuracy (95% CI, 86.89–96.42), 88.61% sensitivity (95% CI, 79.47–94.66), and 98.25% specificity (95% CI, 90.61–99.96), and an AUC of 0.974 (Table S6). The PPV and NPV of mt-uRNA test were 98.59% (95% CI, 92.40–99.96) and 86.15% (95% CI, 75.34–93.47), respectively. Early and late recurrences were defined as recurrence within and after one year of surgery, respectively.^24^ The sensitivity of mt-uRNA for early-recurrent UC was slightly higher than late-recurrent UC (91.18% vs. 86.67%, *p* = 0.79).

## Discussion

Previous studies have documented that urine-derived RNA holds great promise as a category of biomarkers for detection of UC.^18^^,^^19^ Compared to DNA, RNA biomarkers provide a dynamic perspective of disease activity that remains uninfluenced by age-related methylation patterns. In this study, we substantially expanded the training cohort to 688 samples, including 328 UC cases and 360 controls. Then, we applied the refined SVM-RFE algorithm to select the most parsimonious gene set and train the classifier. As reported in the literature, SVM-RFE is particularly effective in identifying a minimal, jointly informative gene set by removing redundant features and prioritizing combinations that maximize classifier performance.^25^^,^^26^^,^^27^ Leveraging these improvements, we identified eight predictive gene markers and developed a urine-RNA test that achieved 93.35% accuracy, 92.28% sensitivity, and 94.05% specificity in a large independent validation cohort of 752 samples.

By reviewing the literature, the eight genes have been reported to be significantly dysregulated in UC. CA9 is consistently elevated in UC tissues compared to benign urothelium and is strongly associated with tumor grade, stage, and prognosis.^28^^,^^29^ Similarly, urinary and tissue studies have demonstrated that IGF2 is upregulated in UC patients, highlighting its potential as a diagnostic and prognostic biomarker.^30^^,^^31^ Elevated expression of MMP12, largely derived from tumor-associated macrophages, has also been linked to aggressive disease and poor outcomes in UCB.^32^ Although previous publications for PPP1R14D overexpression in UC remains limited, complementary analysis of the TCGA bladder cancer cohort using the UCSC Xena platform (http://xena.ucsc.edu/welcome-to-ucsc-xena/) revealed that PPP1R14D is indeed significantly overexpressed in tumor tissues compared to normal counterparts (*p* < 0.001), providing further evidence supporting its potential as a promosing UC biomarker.^33^ CCL18 is a member of the serum-based cytokine family of secreted proteins involved in immunoregulatory and inflammatory processes. Miyake M et al. revealed that adding whole blood to urine from healthy donors resulted in a 5.3-fold increase in urinary CCL18 concentration, suggesting that hemolysis-induced tissue damage triggers CCL18 secretion by immune cells.^34^ Additional immunohistochemical analysis of bladder tumor tissues confirmed that CCL18 was present only in the inflammatory cells located in the stroma. In the present study, by comparing UC patients to individuals with benign urinary tract conditions rather than healthy controls, our results further suggest that elevated CCL18 expression may serve as a potential biomarker for distinguishing hematuria and UTI from UCs. Eriksson P et al. reported that ERBB2 expression varies by molecular/histologic subtype of UC, and many subgroups contain predominantly ERBB2-low/negative tumors.^35^ SGK2 is a serine/threonine kinase that regulates ion transport, cell survival, and signaling pathways relevant to epithelial physiology. Li A et al. reported that reduced SGK2 expression in UCB compared with normal urothelium, highlighting its potential role as a negatively associated gene in urothelial tumorigenesis.^36^ Complementing these tissue-based findings, our study observed that UC patients had lower urinary SGK2 expression than benign disease patients, supporting its potential as a urinary biomarker. SWINGN (also known as LINC00565) has recently been characterized as a functionally relevant long non-coding RNA that interacts with SWI/SNF chromatin remodeling complexes and modulates a tumor-promoting transcriptional program, including activation of GAS6 and other oncogenic genes.^37^ Accumulating evidence indicates that urinary and urinary-exosomal RNAs are stable and reliably preserve lncRNAs, supporting the feasibility of detecting tumor-associated lncRNAs in urine. In this study, we show that SWINGN is expressed at lower levels in urine from patients with UC than in those with benign urinary conditions, suggesting its potential as a differential diagnostic biomarker. The mechanisms underlying this pattern remain unclear and warrant further functional investigation. These genes are applicable for tumor diagnosis and may also facilitate research on tumor progression, with previous studies showing that RNA can be used to predict lymph node metastasis.^38^

The mt-uRNA test achieved an overall accuracy of 93.35% in a large-scale, multicentric prospective cohort, which compared favorably with previous DNA- and RNA-based urine tests.^11^^,^^12^^,^^13^^,^^18^^,^^39^ In subgroup analysis, the mt-uRNA test achieved sensitivities of 100% for stage Ta and 87.74% for stage T1, highlighting its potential utility for early detection of UC. Remarkably, ten of 12 CIS cases were correctly detected with a sensitivity reaching 83.33%. Accurate detection of CIS is essential due to its aggressive nature, impact on prognosis, and detection challenges by routine endoscopic evaluation.^40^ The mt-uRNA test’s high detection rate could pave the way for more effective treatment strategies and improved patient outcomes.

UTUC, despite representing merely 5%–10% of UC, is characterized by approximately 60% of tumors being invasive at the time of diagnosis, making it more aggressive than lower tract urothelial malignancies.^2^^,^^41^ Meanwhile, the diagnosis of UTUC, especially for early-stage tumors, is known to be more challenging due to its occult symptoms. Remarkably, the mt-uRNA test exhibited a sensitivity of 97.30% in UTUC, underscoring its high diagnostic efficacy for this subtype.

Hematuria is the most common sign of underlying urologic malignancy, with UC present in approximately 17% of cases.^42^ However, excessive endoscopy leads to increased financial burden and decreased patient compliance. There were approximately 230,000 unnecessary cystoscopy cases conducted annually in the USA and Europe for patients experiencing hematuria but with relatively low risk of tumor.^43^ The mt-uRNA test achieved a sensitivity of 94.19% at a specificity of 90.63% for detection UC patients among hematuria population. Considering the prevalence of UC among hematuria patients,^42^ the NPV of the mt-uRNA test is adjusted to 98.70%. This high adjusted NPV in a typical screening clinical setting indicates utilizing this mt-uRNA test may greatly assist in minimizing unnecessary cystoscopies. Based on these findings, potential conversion routes for clinical implementation can be envisioned. For instance, in patients presenting with gross hematuria, the mt-uRNA test may serve as an initial, non-invasive screening tool. Individuals with positive results could subsequently undergo confirmatory cystoscopy, thus streamlining diagnostic pathways, improving resource allocation, and reducing the burden of unnecessary invasive procedures.

It is estimated that more than two million patients worldwide are living with UC.^44^ Previous studies proved that invasive detection of endoscopy, incomplete resection during TURBT, and drop metastasis from UTUC always contribute to high rate of recurrence in patients with UC.^5^ Therefore, accurately identifying residual and recurrent tumors through a noninvasive urine test reduced unnecessary or overused endoscopy and second TURBT procedure. The mt-uRNA test demonstrated significant diagnostic advantages in the residual and recurrence subpopulations, with accuracies of 86.89% and 92.65%. Importantly, the mt-uRNA test’s sensitivity for HG tumors reaches 96.15%, suggesting a minimal chance of missing an HG recurrence and a high level of confidence in negative results. Consequently, incorporating this test into routine surveillance could potentially reduce unnecessary diagnostic procedures and TURBTs.

Recent prospective randomized controlled trials have highlighted the significant clinical value of urinary RNA tests in managing UC patients. Lotan Y et al. demonstrated that using Cxbladder triage in patients with low-risk microhematuria led to a 59% reduction in unnecessary cystoscopies without compromising the detection of high-risk cancers.^45^ The UroFollow trial showed that urinary RNA and methylation marker-guided surveillance in low/intermediate-risk NMIBC patients maintained diagnostic accuracy comparable to traditional cystoscopy-based follow-up, suggesting a potential for reducing invasive procedures.^46^ Furthermore, the DaBlaCa-15 trial revealed that alternating the Xpert Bladder cancer monitor with cystoscopy in high-grade NMIBC patients effectively reduced the number of cystoscopies during follow-up, underscoring the test’s utility in guiding clinical decision-making.^47^

In this study, our mt-uRNA test demonstrated robust performance in both the early detection and recurrence monitoring of UCs. These findings, together with evidence from recent prospective trials of urinary RNA assays, highlight the growing role of urinary RNA testing in optimizing diagnostic and surveillance strategies. With further validation in large-scale prospective studies, the mt-uRNA test might have the potential to be integrated into routine clinical practice, thereby improving patient management while reducing reliance on invasive procedures.

### Limitations of the study

The current study has certain limitations. First, the number of patients with early-stage disease, particularly those of CIS and Ta stages, is relatively small. Additional studies will be needed in the future to address this limitation. Second, the urine samples were collected at a single preoperative time point in the surveillance population of UC and the evaluation of the mt-uRNA test performance was conducted via a cross-sectional analysis, which still requires postoperative continuous monitoring to assess the performance of mt-uRNA test for disease relapse monitoring, especially for conducting survival analysis to evaluating whether a test can effectively predict the duration of relapse-free status and predict outcome, as well as conduct multivariate analysis in conjunction with other prognostic factors. Then, despite using specialized urine preservatives, samples must be processed promptly and prolonged storage of urine will affect RNA stability. Besides, there is a potential misclassification for UTI and urolithiasis cases, with a false positive rate of 6 in 78 and 1 in 7 cases, respectively. Nevertheless, the advantages of the mt-uRNA test over traditional approaches needs to be further validated in future prospective studies.

## Resource availability

### Lead contact

Further information and requests for resources and reagents should be directed to and will be fulfilled by the lead contact, Qinghua Xu (qinghua.xu@canhelpgenomics.com).

### Materials availability

This study did not generate new unique reagents.

### Data and code availability

• The datasets used in this study from the Fudan University Shanghai Cancer Center and other collaborating centers are not publicly available due to patient privacy concerns. To request access to the data, please contact the lead contact, who will connect you with the responsible researcher at the corresponding center. Data will be accessible only if the Ethics Committee of each center where the data were collected grants permission. Therefore, the requester must describe the project for which data access is requested, detailing the objectives and data management plan. Data access will be considered for research purposes and non-commercial use only. To ensure patient privacy, access to personally identifiable information or sensitive clinical information (including medical histories) will not be provided, and requests for data access must rigorously adhere to the consent agreements established with study participants.
• This paper does not report original code.
• Any additional information required to reanalyze the data reported in this paper is available from the lead contact upon request.

## Acknowledgments

We thank all the participants for their contribution to this study. This work was supported by grants from the 10.13039/501100001809National Natural Science Foundation of China (no. 82172741) and Joint Breakthrough Project for New Frontier Technologies of the 10.13039/501100008750Shanghai Hospital Development Center (no. SHDC12022105). Graphical abstract was created with https://BioRender.com.

## Author contributions

D.Y., X.Q., G.L., and Q.X.: conception, design, and supervision. H.X., S.W., D.X., Y.W., Z.L., J.C., and Q.X.: biomarker evaluation and assay development. H.X., S.W., D.X., Z.X., Q.Z., Q.W., B.H., Z.M., F.L., S.L., J.Y., S.Z., and L.D.: acquisition of data, analysis, and interpretation of data. H.X., S.W., and D.X.: drafting of the manuscript. D.Y.: funding acquisition. Y.W., Z.L., J.C., and Q.X.: administrative, technical or material support. All authors had full access to all the data in the study and had final responsibility for the decision to submit for publication.

## Declaration of interests

Y.W., Z.L., J.C., and Q.X. are current employees of Canhelp Genomics Co., Ltd. The remaining authors declare that they have no competing interests.

## STAR★Methods

### Key resources table

**Table:** 

REAGENT or RESOURCE	SOURCE	IDENTIFIER
**Biological samples**		

Human urine samples	This paper	N/A

**Chemicals, peptides, and recombinant proteins**		

Ethanol	Yonghua Chem	CAS#64-17-5
Chloroform	Yonghua Chem	CAS#67-66-3

**Critical commercial assays**		

Urine Collection and Preservation Tube	Canhelp Genomics	CAT#G01000501
Urine Exfoliated Cells RNA Isolation Kit	Canhelp Genomics	CAT#G01000402
Gene Expression Test System for Human Urothelial Cancer Detection	Canhelp Genomics	CAT#G05000101

**Software and algorithms**		

R version 4.3.1	The R Project for Statistical Computing	https://www.r-project.org
ToppGene	ToppGene Suite	https://toppgene.cchmc.org/
UCSC	UCSC Xena platform	http://xena.ucsc.edu/welcome-to-ucsc-xena/

**Other**		

Real-Time PCR System	Tianlong	Gentier 96E

### Experimental models and study participant details

#### Comprehensive training dataset and biomarker identification

Building on our earlier research, we initially identified a 32-gene panel for the detection of UCB using a training set of 211 urine samples, and subsequently confirmed its reliability in a validation cohort of 317 urine samples.^20^ This was followed by the development of an eight-gene panel for the noninvasive detection of UTUC, which we validated using an independent multicentric cohort consisting of 160 samples.^22^ To further enhance biomarker discovery for noninvasive detection of UC, we have pooled all available samples from previous studies to form a comprehensive training set. This upgraded dataset serves as a robust foundation for biomarker discovery and development of diagnostic test.

We utilized the recursive feature elimination algorithm for feature selection, and the linear SVM algorithm for classification model fitting. The 10-fold CV process was used for internal validation within the training set. The final model coefficients were determined with the whole training set, and used to calculate a risk score and classify the UC status of new specimens.

#### Inter-laboratory/inter-operator reproducibility

This test aimed at assessing the inter-laboratory/inter-operator variability of PCR results and risk scores of the mt-uRNA test to ensure the reliability of multicentric validation study. Urine samples from 9 UC patients, were pooled and distributed to three operators in different laboratories. Each operator tested the samples for five consecutive days, with all results subsequently submitted to a coordinating operator for centralized analysis to determine the inter-laboratory and inter-operator reproducibility of cycle threshold (Ct) values for each target and corresponding risk scores.

#### Prospective validation study and participants

To further validate the performance of the mt-uRNA test in clinical settings, we conducted a prospective, multicenter study according to the Standards for Reporting of Diagnostic Accuracy guidelines.^48^ This study was performed in accordance with the Declaration of Helsinki and was carried out in three leading Chinese medical centers: Fudan University Shanghai Cancer Center, Zhejiang Provincial People’s Hospital and Sir Run Run Shaw Hospital affiliated with the Zhejiang University School of Medicine between June 2022 and September 2023. Institutional review board approval was obtained from all participating centers and all participants provided written informed consent. Individuals were considered eligible if they presented with hematuria, irritative voiding symptoms or dysuria, abnormal urine cytology, and/or suspicious mass detected during imaging examinations. The exclusion criteria included prior cancer therapy within the past month, the absence of cystoscopy/histological results, and withdrawal of consent or drop out. The corresponding clinical characteristics of patients’ age and gender were also recorded. The available results of routine clinical urine-based tests (including urine cytology, the NMP22 test, and UroVysion FISH) were retrieved for the comparative analysis.

### Method details

#### Sample collection and processing

Urine for testing was collected from the first miction in the morning before standard-of-care treatments, such as TURBT, cystectomy, or other surgeries, were performed. Voided urine (approximately 50–100 mL) was collected and stabilized using the Urine Collection and Preservation Tube (Canhelp Genomics, Hangzhou, China) and kept at 2–8°C. All patient samples were centrifuged at 1,500 rpm for 10 min within 72 h of collection, and urine sediment pellets were retained for RNA extraction and reverse transcription per the manufacturer’s protocol, as previously described.^22^ The RNA expression profiles were analyzed using RT-PCR on Gentier 96E Real-Time PCR system (Tianlong, Xi’an, China). The *GAPDH* gene, shown relatively stable expression levels in collected urine samples, is used as an endogenous control.^20^ Urine samples with *GAPDH* Ct values above 30 were excluded from the study. For all valid samples, the relative expression levels of the target genes were normalized by ΔCt method, where ΔCt = Ct (target gene) - Ct (*GAPDH*). These ΔCt values are subsequently incorporated into a pre-trained SVM model, from which the final risk score is automatically calculated using R statistical software.

### Quantification and statistical analysis

All data were analyzed and presented using descriptive statistics, including mean, range, and proportions. Accuracy, sensitivity, specificity, PPV and NPV with the corresponding 95% CIs were calculated to assess the performance of the biomarker test. The AUC was calculated to evaluate the discriminatory ability of the risk score. For continuous variables, intergroup comparisons were conducted using the Wilcoxon Rank-Sum test. For categorical variables, intergroup comparisons were conducted using Pearson’s Chi-squared test and Fisher’s exact test, as appropriate. The statistical analysis was conducted by R (version 4.3.1). Two-tailed tests were employed for all comparisons, and *p* values below 0.05 were deemed statistically significant.

## Supplemental information

Document S1. Figures S1

Table S1. Functional enrichment analysis of eight genes, related to Table 1

Table S2. Patient characteristics in recurrent subpopulation, related to Table 2

Table S3. Diagnostic performance of the mt-uRNA test versus cystoscopy/histopathology in overall population, related to Figure 2A

Table S4. Diagnostic performance of the mt-uRNA test versus cystoscopy/histopathology in hematuria subpopulation, related to Figure 3A

Table S5. Diagnostic performance of the mt-uRNA test versus cystoscopy/histopathology in residual subpopulation, related to Figure 3B

Table S6. Diagnostic performance of the mt-uRNA test versus cystoscopy/histopathology in recurrent subpopulation, related to Figure 3C
