# Supplementary material for: Development and prospective validation of a multitarget urine RNA assay for noninvasive detection of urothelial carcinoma
Source: iScience. 2025 Nov 19;28(12):114154. doi: 10.1016/j.isci.2025.114154 (PMC12744290; doi:10.1016/j.isci.2025.114154)
Supplement: Document S1. Figures S1 [file mmc1.pdf]

iScience, Volume *28*

Supplemental information

Development and prospective validation of a multitarget urine RNA assay for noninvasive detection of urothelial carcinoma

Hua Xu, Shuai Wang, Dingwei Xue, Zhihui Xu, Qi Zhang, Qijun Wo, Banggao Huang, Zujie Mao, Feng Liu, Shuixin Lou, Jie Yuan, Shibin Zhu, Lifeng Ding, Yixin Wo, Zhengzhi Luo, Jinying Chen, Qinghua Xu, Gonghui Li, Xiaolong Qi, and Dingwei Ye

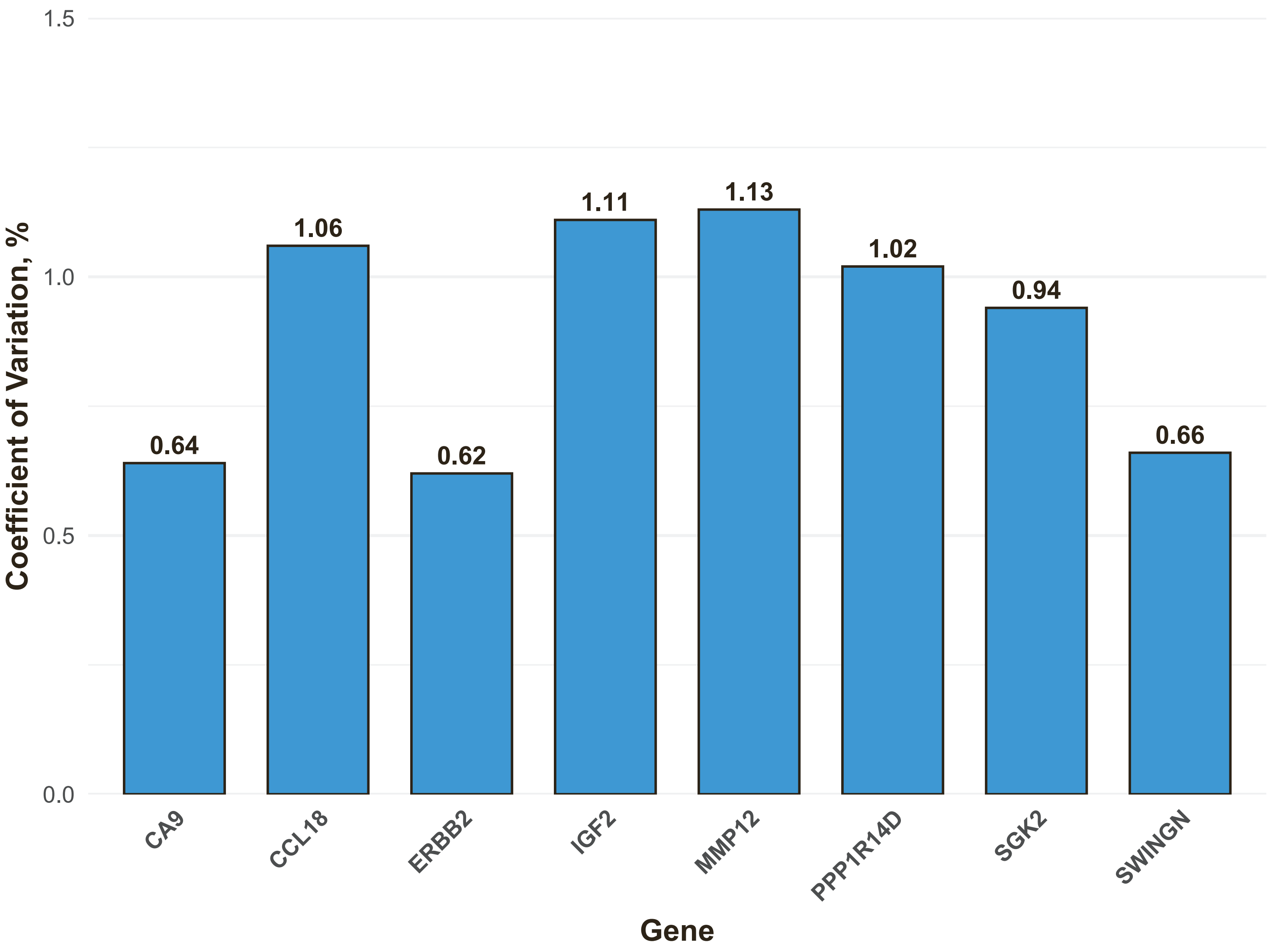

Figure S1. Assessment of reproducibility for each target gene, Related to STAR Methods.

Bar graphs show the coefficient of variation (%) of Ct values for each target gene, calculated from measurements by three operators in three laboratories over a course of five days. These values reflect inter-laboratory and inter-operator variability and are presented as descriptive statistics from the reproducibility study.
